# Editorial: Design of extended networks for tuning functionality of materials

**DOI:** 10.3389/fchem.2026.1866734

**Published:** 2026-06-01

**Authors:** Eugeny V. Alexandrov, Alexey E. Kuznetsov, Yuanzuo Li

**Affiliations:** 1 Bauman Moscow State Technical University, Moscow, Russia; 2 Federico Santa María Technical University, Valparaíso, Chile; 3 Northeast Forestry University, Harbin, China

**Keywords:** advanced synthetic techniques, design of materials, extended structures, structural patterns and descriptors, tuning properties

## Introduction

Artificial intelligence technologies and mathematical modeling are integrating into chemistry and materials science, providing sophisticated tools capable of extracting complicated “composition-structure-property” relations. These tools reduce uncertainties related to experimental synthesis and provide a digital background for the design of materials with specific properties. The design of crystalline (extended) structures contrasts with organic chemistry due to the low predictability of the former. Furthermore, periodic structures have additional stereochemical, topological, and symmetrical constraints that are not yet fully understood. Novel computational approaches involved in the systematization of information and new descriptors derived from the structures of extended networks uncover patterns and regularities that dictate the structural organization and functionality of materials.

In this Research Topic, four studies focused on designing extended networks tailored to tune the functionality of materials, including octahedral and icosahedral networks, covalent organic frameworks, zeolites as adsorbents, and planar tetracoordinate carbon molecules as catalysts. A summary of the main results of these works is presented below.

### Computational measures of irregularity and molecular descriptors

Extended networks, such as metal-organic coordination polymers, high-performance polymers, covalent organic frameworks, and hydrogen-bonded organic frameworks, have high structural complexity, which complicates their structural description and the search for correlations between structural descriptors and properties. Symmetry and topology indexes decipher the bonding pattern and structural properties of networks. The greatest size of irregularity in a network is still an open question. Therefore, irregularity indices are in demand in chemistry. Zhang et al. proposed new computational schemes for irregularity indices of octahedral OTn and icosahedral ISn networks. Higher values were found for ISn networks across a range of network complexities. Irregularity indices are useful in the estimation of entropy, enthalpy, and phase transitions. The results offer pathways for designing new, more complex networks.

### Topological entropy characterization and graph energy prediction

Computing the energy characteristics of extended structures using quantum chemical methods requires the use of powerful supercomputers. These characteristics determine mechanical properties, chemical stability, solubility, and so on. Empirical mathematical relations can facilitate energy computation by orders of magnitude. However, they can only be determined for a series of topologically related structures. These mathematical models were proposed by Raza et al. to predict graph energy for Marta-covalent organic frameworks (COFs). COFs are composed of polycyclic aromatic hydrocarbons with contorted hexabenzocoronene (c-HBC) at their core. The c-HBC unit, copolymerized with pyrene-2,7-diboronic acid, forms Marta-COF-1. Similarly, Marta-COF-2 was obtained by copolymerization of c-HBC with benzene-1,4-diboronic acid. Mathematical expressions for topological descriptors were derived from index functions combined with the edge classes, including geometric, harmonic, and Zagreb descriptors. Shannon’s entropy method used a structural information function to obtain entropy quantities. Using correlation analysis, the relationship between topological descriptors and graph energy for two series of Marta-COFs was identified. The predicted energy of the Marta-COFs, based on degree descriptors, was predicted with high precision, showing its usability in estimating graph energy values in complex Marta-COFs.

### Assessment of interaction energies using topological and spectral entropies

Zeolites are microporous minerals of aluminosilicate nature widely used as adsorbents and catalysts in gasoline production, medicine, ion exchange, and sensors. Peter et al. focused on developing generalized expressions of degree- and degree-sum-based measures of graphs for the AWW zeolite structure. The complexity of the AWW zeolite was estimated using these descriptors. Furthermore, mathematical models of the relationships between measured entropies and molecular interaction energies provided insight into the energy and structural characteristics of zeolites. Eigenvalues of the structures provided the basis for the calculation of the HOMO–LUMO gap, global chemical reactivity descriptors, and spectral properties. Thus, the quantitative structure–property relationship approach is effective for predicting the stability and properties of structurally similar zeolite materials.

### Catalytic hydrogenation of alkyne with planar tetracoordinate carbon

Planar tetracoordinate carbon (ptC) has received significant theoretical and experimental interest. ptC molecules are experimentally identified through the generation of clusters in the gas phase under laser irradiation, followed by isolation via time-of-flight mass spectrometry. Knowledge of ptC structures and the mechanisms by which they interact with organic molecules in chemical reactions could lead to new approaches in chemical synthesis. Malhan and Thirumoorthy studied the potential of the ptC molecule CAl_3_MgH_2_¯ in the catalysis of hydrogenation reactions based on quantum chemical computations for the first time. CAl_3_MgH_2_¯ was found to be effective in converting alkynes to alkenes and alkenes to alkanes under similar conditions. Significantly, a detailed reaction mechanism was proposed for the catalytic process of hydrogenation of alkyne and alkene using the CAl3MgH2¯ catalyst. These mechanistic insights into the hydrogenation of alkyne and alkene on CAl_3_MgH_2_¯ will assist in designing effective main-group metal-based catalysts for hydrogenation reactions.

In summary, the authors’ findings within this Research Topic provide a mathematical background for the search and design of extended architectures with promising adsorption and catalytic properties. We thank the authors, reviewers, and editors for their contributions to this Research Topic.

